# A new species of *Schrankia* Hübner, 1825 from China (Lepidoptera, Erebidae, Hypenodinae)

**DOI:** 10.3897/zookeys.242.3856

**Published:** 2012-11-15

**Authors:** Oleg Pekarsky

**Affiliations:** 1 H-1068 Budapest, Felsőerdősor u. 16-18, Hungary

**Keywords:** Lepidoptera, Erebidae, *Schrankia*, new species, China

## Abstract

A new species of the genus *Schrankia* Hübner, 1825,* S. pelicano* **sp. n.** is described from Sichuan, China. A diagnostic comparison is made with *Schrankia taenialis* (Hübner, [1809]) and *Schrankia separatalis* (Herz, 1904); illustrations of the male holotype and its genitalia are provided. A checklist of the genus with synonyms is given.

## Introduction

*Schrankia* Hübner, 1825 is a widespread genus of Erebidae, being distributed in the Northern hemisphere, the Oriental and Australasiatic regions. It is represented by three species in Europe (Fibiger et al. 2010, Sinev 2008), three species in Africa and a single species on the Seychelles (Hacker 2004), twelve species in eastern and south-eastern Asia (Kononenko et al. 1998, Kononenko and Han 2007, Inoue 1979, Sugi 1982, Holloway 2008, Galsworthy 1997, Kendrick 2002). A considerable number of species live in Australasiatic region (Holloway 1977, Robinson 1975, Evenhuis 2007); three species in Central America one of which, *Schrankia macula* (Druce, 1891), also inhabits North America (Lafontaine and Schmidt 2010); there are only two valid taxa remaining in Hawaii after the remarkable revisionary work of Medeiros et al. (2009). The European taxon, *Schrankia intermedialis* Reid, 1972 is treated by Fibiger et al. (2010) as an interspecific hybrid of *Schrankia costaestrigalis* and *Schrankia taenialis*. The genus has been reported twice from Hong Kong (Galsworthy 1997, Kendrick 2002) but has not been mentioned in any Chinese literature (Chen et al. 1991, Chen 1999, Li-zhong 2005); this is the first record of the genus from south-west China.

## Systematic part

### Genus Schrankia

Hübner, 1825

http://species-id.net/wiki/Schrankia

Figs 3 , 8

Pyralis taenialis Hübner, [1809]. Type-species.

#### Remarks.

The genus *Schrankia* is characterized by the slender body and narrow, weakly sclerotized wings, light-brown ground color of the forewing and abdomen, long, straight labial palps, which are three to four times as long as the diameter of the eye (Zimmerman 1958) and the absence of ocelli. In the male genitalia (Fig. 8), uncus long, slightly curved; valva elongated with acute apex, bearing three well-developed processes in the middle; juxta X-shaped, composed of two well-sclerotized, bent bars; aedeagus thin, elongated, slightly curved with club-like caecum. The externally often confusingly similar species of the genus *Hypenodes* (type-species *Hypenodes humidalis* Doubleday, 1850) have a smaller and thinner body, narrow wings, grey or brownish forewing ground color, upcurved labial palps; ocelli also are absent. The configuration of the male genitalia is very uniform throughout the genus, having a very simple, long, narrow valva with small and thin processes at the base, and a short, wide aedeagus with a tapered caecum.

### Schrankia pelicano sp. n.

urn:lsid:zoobank.org:act:033BC935-14EC-4A82-B753-15C530773B3D

http://species-id.net/wiki/Schrankia_pelicano

Figs 1, 2 , 5–7

#### Type material.

**Holotype** male. China, Sichuan, 29°52.808'N, 102°50.240'E, near Ying Jing, bamboo forest, 700 m, 4.IV.2011, leg. Floriani; slide No.: OP1429m (coll. O. Pekarsky, deposited in the HNHM Budapest). **Paratypes.** China, Sichuan: 4 ♂♂, with same data as the holotype (coll. A. Saldaitis, Vilnius); 1 ♂ (coll. O. Pekarsky); 1 ♂ (coll. W. Speidel, Munich).

#### Etymology.

The species name refers to the resemblance of the opened male genitalia to a pelican.

#### Diagnosis. 

The new species possesses a number of the diagnostic characters of the genus *Schrankia* (e.g., absence of ocelli; long, straight palpi; wide valval base; presence of medial complex of processes; thin, elongated aedeagus). The autapomorphic features of *Schrankia pelicano* are the very long palpi, being much longer than in other species of *Schrankia*, the biarticulate uncus, the extremely large sacculus, which is almost equal in size and similar in shape to the distal half of the valva. Considering the diagnostic characters mentioned previously, the new species is placed into *Schrankia*, but its generic position could change through a much needed revision of the entire genus. Among *Schrankia*, the new species is closest to the Oriental group of species known from Korea and Japan, *Schrankia separatalis*, *Schrankia dimorpha*, *Schrankia kogii*, *Schrankia masuii* and *Schrankia seinoi*. This species-group is characterized by the flat, thin, weakly sclerotized (almost transparent) distal half of valva, which has a rounded apex, wider and more strongly sclerotized haunch-like valval base, the well-developed apical saccular projection, and the less broadened caecum. The numerous autapomorphic features of the male genitalia of the new species (Figs 5–7) make it difficult to determine its closest relative within *Schrankia*. The comparison is provided here with *Schrankia taenialis* (Fig. 8), the type-species of the genus and *Schrankia separatalis* (Fig. 9), the most similar representative of the Oriental group of species. *Schrankia pelicano* is similar externally to *Schrankia taenialis* and *Schrankia separatalis* (Fig. 4) but has longer labial palpi, narrower, more elongated forewing with a less acute apex and straight, almost parallel crosslines without undulations or dentations. The male genitalia of *Schrankia pelicano* differ from those of the related species by the long and narrow, subapically constricted valva, very large sacculus, digitiform ampulla, and basally clavate uncus with a long, narrow, acute distal part. *Schrankia taenialis* (Fig. 8) has a narrow valva with an acute apex, a large and broad ampulla and a long uncus. The genitalia of *Schrankia separatalis* have, in comparison with *Schrankia pelicano*, short and wide valva with small sacculus and much thicker, continuously curved uncus having half-cylindrical cross-section (Fig. 9).

**Figures 1–4.**
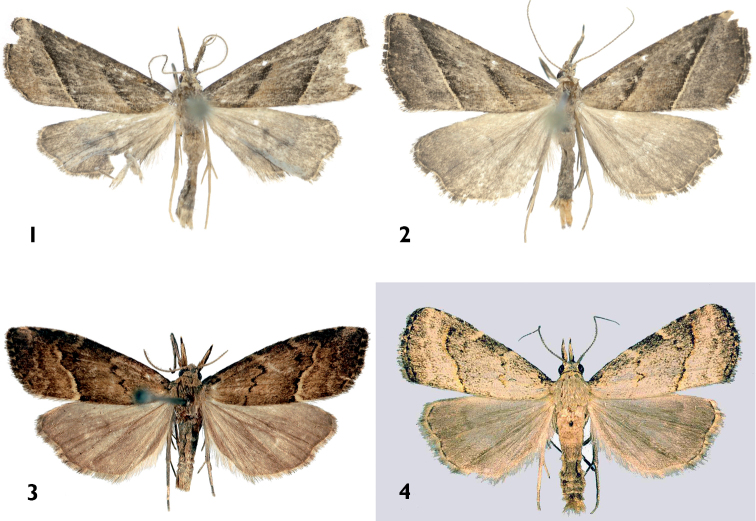
Adults. **1–2** *Schrankia pelicano* sp. n. **1** holotype male, China **2** paratype male, China **3** *Schrankia taenialis* male, Hungary **4** *Schrankia separatalis* male, Korea.

#### Description.

Male (Figs 1, 2). Wingspan 16–17 mm, length of forewing 8 mm. Head, thorax and abdomen ochreous grey; ocelli absent; tongue well developed; palpi very long (almost 5 times longer than diameter of eye), straight, 2^nd^ segment (with appressed scales) about 2.8 times longer than 3^rd^ segment, which is porrect; antenna with fine ciliation ventrally along full length, basal joint without pectination. Forewing elongate, narrow with acute apex, outer margin with rounded termen; ground color grey brown; costa straight with five milk-white patches; basal line barely visible, semicircular; antemedial line black, straight, oblique, curved upward near costa; postmedial line black, straight, oblique, extending from 2/3 from base on hind margin of wing to 9/10 from base on costal margin, edged on outer side by narrow yellow postmedial fascia; subterminal line faint, pale, irregular, parallel to outer margin of wing; terminal line black, most prominent between wing veins; cilia yellow at base with dark medial line, grey distally. Hindwing ochreous grey, discal spot grey, terminal line black; cilia pale yellow at base, grey distally. Abdomen slender, long. Female unknown.

**Figures 5–9.**
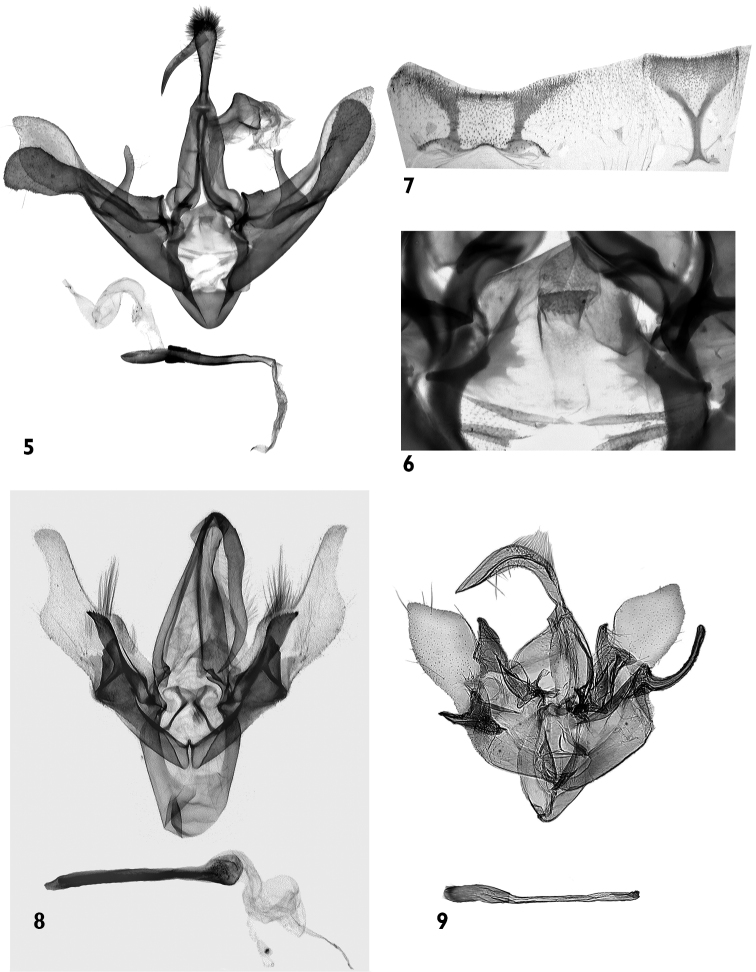
Male genitalia. **5–7** *Schrankia pelicano* sp. n., holotype, China, slide No. OP1429m **5** clasping apparatus and aedeagus with vesica inverted 6 juxta (medially enlarged) **7** 8^th^ abdominal segment **8** *Schrankia taenialis* Hungary, slide No. 10059 RL **9** *Schrankia separatalis* Korea, slide V. Kononenko.

**Male genitalia** (Figs 5–7). Uncus biarticulate, consisting of clavate and setose main part and long, narrow, subapically curved, bill-like extension with pointed tip; tegumen narrow, as long as vinculum; scaphium well sclerotized, distally dilated; subscaphium membranous; juxta large, triangular, wider at base, weakly sclerotized (almost transparent), with two narrow, strongly sclerotized lateral plates with serrated inner edges (Fig. 6); vinculum strong, cup shaped. Valva elongated, conspicuously constricted subapically; cucullus almost rounded with acute tip; corona absent; sacculus very large, elongated, with very long, heavily sclerotized, distally dilated saccular extension, almost as long as distal half of valve; clavus unspecialised; clasper/ampulla complex large, sclerotized, setose, cuneate with broad base and finely pointed apex. Aedeagus tubular, thin and gracile, finely undulate. Vesica relatively short (shorter than aedeagus), thinly tubular (as broad as average width of aedeagus), with fine granulose scobination throughout.

#### Distribution.

The species is known only from the type-locality, South-West China, Province Sichuan.

##### Checklist of *Schrankia*

**Europe**

*Schrankia taenialis* (Hübner, [1809]) TL: Europe

syn. *albistrigatis* Haworth, 1809 [TL: Britain]

*Schrankia costaestrigalis* (Stephens, 1834) TL: Wittlesea Mere, Swaffham, Norfolk

syn. *exsularis* Meyrick, 1888 TL: New Zealand, Taranaki

syn. *triangulalis* Hudson, 1923

syn. *costistrigalis* Dannehl, 1925 [TL: Italy]

syn. *lugubralis* Dannehl, 1925 TL: South Tirol, Italy

syn. *monotona* Lempke, 1949 [TL: Holland]

syn. *unicolor* Lempke, 1949 [TL: Holland]

syn. *virgata* Lempke, 1966 [TL: Holland]

syn. *hartigi* Berio, 1991 TL: Sardinia

*Schrankia balneorum* (Alphéraky, 1880) TL: N. Caucasus

syn. *bosporella* Budashkin & Klyuchko, 1990 TL: Crimea

**Africa**

*Schrankia solitaria* Fletcher, 1961 TL: Ruwenzori, Mahoma River [Uganda]

*Schrankia microscopica* (Berio, 1962) TL: Aldabra Islands [Seychelles]

*Schrankia namibiensis* Hacker, 2004 TL: Namibia, Brandberg, Am Königstein

*Schrankia scoparioides* Hacker, 2004 TL: Namibia, Brandberg, Hungarob-valley

**Asiatic region**

*Schrankia obstructalis* (Walker, [1866]) TL: Sarawak [Malaysia]

*Schrankia croceipicta* (Hampson, 1893) TL: Ceylon, Pundaloya

syn. *croceipicta aegrota* Berio, 1962; 179, TL: Seychelles, Mahe B., Vallon

*Schrankia aurantilineata* (Hampson, 1896) TL: Ceylon

*Schrankia separatalis* (Herz, 1904) TL: Korea

syn. *squalida* Wileman & South, 1917; 28, TL: Japan

*Schrankia dimorpha* Inoue, 1979 TL: Kagawa Pref., Shinoe, Fudodaki [Japan]

*Schrankia kogii* Inoue, 1979 TL: Hokkaido, Shintoku, Kuttari

*Schrankia masuii* Inoue, 1979 TL: Kagawa Pref., Shinoe, Oyashiki [Japan]

*Schrankia seinoi* Inoue, 1979 TL: Amami-Oshima Is., Sumiyoson [Japan]

*Schrankia bilineata* Galsworthy, 1997 TL: Hong Kong

*Schrankia pelicano* sp. n. TL: China, Sichuan

*Schrankia bruntoni* Holloway, 2008 TL: Ulu Temburon, Brunei

*Schrankia dusunorum* Holloway, 2008 TL: Sabah, Ulu Dusun, 30mls W of Sandakan [Malaysia]

*Schrankia spiralaedeagus* Holloway, 2008 TL: Sarawak, Gunong Mulu Nat. Park [Malaysia]

**Australasiatic region**

*Schrankia calligrapha* Snellen, 1880 TL: New Hebrides, Aneityum, Red Crest, 3 km NE of Anelgauhat

*Schrankia taona* (Tams, 1935) TL: Samoa, Savaii

*Schrankia capnophanes* (Turner, 1939) TL: Tasmania, Mt. Wellington

*Schrankia dochmographa* Fletcher, 1957 TL: Solomon Is., Rennell I., Hutuna

*Schrankia furoroa* Robinson, 1975 TL: Fiji, Rotuma, Furoroa

*Schrankia vitiensis* Robinson, 1975 TL: [Fiji]

*Schrankia boisea* Holloway, 1977 TL: New Caledonia, Port Boise

*Schrankia cheesmanae* Holloway, 1977 TL: New Hebrides, Aneityum, Red Crest, 3 km NE of Anelgauhat

*Schrankia daviesi* Holloway, 1977 TL: Norfolk Is., N. Mission Road

*Schrankia erromanga* Holloway, 1977 TL: New Hebrides, Erromango I., Nouankao Camp

*Schrankia karkara* Holloway, 1977 TL: New Guinea, Karkar I., Dampier I.

*Schrankia nokowula* Holloway, 1977 TL: New Hebrides, Sanot, Mt. Tabwemasana, Nokowula

*Schrankia nouankaoa* Holloway, 1977 TL: New Hebrides, Erromango I., Nouankao Camp

*Schrankia tabwemasana* Holloway, 1977 TL: New Hebrides, Santo, Mt. Tabwemasana, Nokowula

*Schrankia tamsi* Holloway, 1977 TL: Samoa, Upolu I., Mt. Vaea

**Neotropical region**

*Schrankia macula* (Druce, 1891) TL: Panama, Chiriqui

*Schrankia flualis* (Schaus, 1916) TL: Panama, Trinidad River

*Schrankia musalis* (Schaus, 1916) TL: Panama, Trinidad River

**Oceanian region**

*Schrankia altivolans* (Butler, 1880) TL: Hawaii, Mauna Loa

syn. *simplex* (Butler, 1881) TL: Hawaii

syn. *oxygramma* (Meyrick, 1899) TL: Kaua, Kaholuamano [Hawaii]

syn. *sarothrura* (Meyrick, 1899) TL: Hawaii, Ola

syn. *arrhecta* (Meyrick, 1904) TL: Hawaii, Mt. Waimea

*Schrankia howarthi* Davis & Medeiros, 2009 TL: Hawaii

## Supplementary Material

XML Treatment for Schrankia

XML Treatment for Schrankia pelicano
